# Severely reduced physical performance is already present at the time of admission for stem cell transplantation

**DOI:** 10.1136/bmjsem-2024-001907

**Published:** 2024-06-13

**Authors:** Ronja Beller, Gabriele Gauß, Oliver Basu, Stefan Schönberger, Michaela Höfs, Dirk Reinhardt, Miriam Götte

**Affiliations:** 1 Clinics for Paediatrics III, Department of Paediatric Haematology/Oncology, West German Cancer Centre, University Hospital Essen, Essen, Germany; 2 West German Cancer Centre, University Hospital Essen, Essen, Germany

**Keywords:** Oncology, Exercise, Children, Rehabilitation, Sports rehabilitation programs

## Abstract

**Objectives:**

Paediatric patients with cancer undergoing allogeneic haematopoietic stem cell transplantation (allo-HSCT) face a high risk for life-threatening infections and transplant-related complications. Therefore, these children should be in the best possible physical condition beforehand. The study aims to evaluate the fitness status before allo-HSCT and identify correlations between fitness, quality of life and fatigue, clinical data, and previous exercise sessions.

**Methods:**

Paediatric patients with cancer ≥4 years old, treated with allo-HSCT, were recruited for the ANIMAL trial ("Effects of a low vs. moderate intense exercise program on immune recovery during paediatric allo-HSCT.", DRKS ID:DRKS00019865). Assessed at admission for HSCT were (1) clinical and anthropometric data, (2) motor performance (strength, endurance and balance) and (3) psychological parameters. Values were compared with published reference values (normative data from the literature) of healthy children, and correlation analyses were conducted.

**Results:**

22 paediatric patients undergoing pre-allo-HSCT (23% female, 9.4±4.5 years, 73% leukaemia) exhibited substantial reduced differences in all motor performance parameters, with up to −106%±98 (mean difference to reference value) in static stance, −37%±45 in sit-to-stand, −52%±16 in leg extension and −48%±22 in hand grip strength compared with reference values. Correlations were observed among age and fitness parameters, the number of inpatient days and fatigue, and many previous exercise sessions correlated with better hand grip strength.

**Conclusion:**

These results indicate a poorer fitness status in children before HSCT compared with healthy children, recommending the need for structured exercise programmes for children undergoing HSCT. Differently directed correlations between age/body mass index and endurance/strength and between exercise sessions and strength show the importance of individualised training recommendations and the effect of training.

**WHAT IS ALREADY KNOWN ON THIS TOPIC**

- Physical fitness in patients with cancer correlates with clinical outcomes. Children with cancer already show significant motor deficits at diagnosis, but especially during chemotherapy, which impair quality of life and reduce resilience. Data on fitness levels before allogeneic haematopoietic stem cell transplantation (allo-HSCT) as a highly intensive therapy are unavailable.

**WHAT THIS STUDY ADDS**

- Paediatric patients at admission for HSCT already show a significantly lower fitness level than reference data from the literature. Previous exercise sessions correlate with higher muscle strength values.

**HOW THIS STUDY MIGHT AFFECT RESEARCH, PRACTICE OR POLICY**

- The study supports the need for supervised individual exercise programmes in patients with childhood cancer undergoing HSCT to increase physical activity and improve functional status before admission.

## Introduction

In Germany, 3005 allogenic haematopoietic stem cell transplantations (allo-HSCT) occurred in 2022, with 9% involving paediatric patients.^1^ These treatments are highly intense, have many side effects and are associated with a high risk for infections and transplant-related complications. Patients often suffer from long-term sequelae.^2–7^ Furthermore, the long and isolated hospitalisation contributes to sedentary behaviour related to adverse symptoms^8^ and is particularly burdensome for children and youth.

Patients lose their aerobic capacity, muscle strength and health-related quality of life (HRQoL) following transplantation.^9 10^ Even before allo-HSCT, most children have a long hospital history, with many inpatient stays and some with relapsed cancer diagnoses. How fit they are is still unknown. First, studies reveal that exercise programmes in paediatric oncology are a promising way to prevent or minimise these consequences and yield positive effects on body composition, flexibility, cardiorespiratory fitness, muscle strength and HRQoL.^11–13^ Therefore, there might be correlations between exercise sessions and fitness levels pre-HSCT. While studies in adult patients undergoing HSCT reveal positive effects of exercise programmes on physical functioning, medical outcomes, with baseline fitness seeming to be protective against mortality, and psychological parameters,^14–16^ as well as a better overall survival for pre-HSCT fitness status,^17^ data in paediatric oncology remain limited.^18^ Some studies indicate that exercising with children may be beneficial, safe and feasible.^19–21^ Studies in adults and in children that compared the initial fitness level before HSCT versus after HSCT of unfit and fit patients suggested that unfit patients have a greater benefit from exercise programmes during HSCT.^22 23^ For paediatric patients, there is little knowledge about fitness levels and what the content of exercise programmes might look like. Whether the fitness level correlates with psychological factors and whether participation in previous exercise programmes influences the fitness level is still unknown.

Therefore, this study aims to evaluate the fitness status before allo-HSCT and to identify correlations between clinical data and anthropometric data, motor performance and psychological parameters of paediatric patients before allo-HSCT.

## Methods

### Study design and participant recruitment

The presented data are baseline data from the exploratory randomised controlled ANIMAL trial, ‘Effects of a low versus moderate intense exercise programme on immune recovery during paediatric allo-HSCT’ (DRKS00019865), which aims to investigate, among others, the effects of an exercise programme on immune recovery. Patients, aged 4–21 years, treated in paediatric oncology with the diagnosis of a haemato-oncological disease and undergoing allo-HSCT were included between January 2020 and March 2023. Exclusion criteria were other diseases, contraindications for physical exercise assigned by the oncologist and an inability to follow training protocol. The language was not an exclusion criteria. Patient recruitment was done through regular meetings and medical consultations, ensuring that all patients were reached. The Ethics Committee of University Hospital Essen, Germany, approved the study protocol according to the ethical guidelines of the Declaration of Helsinki (19–8789-BO). Written consent was given by legal guardians and patients (over 8 years old). Patients had different pretreatments and protocols in different hospitals before HSCT and had, therefore, participated in different physiotherapeutic measures and/or exercise interventions before baseline assessments.

### Assessments and outcomes

Baseline assessments took place after admission to the transplant ward in the patient’s room, 1 or 2 days before starting the conditioning regime. They included the following parameters: (1) clinical and anthropometric data, (2) motor performance and (3) psychological parameters. Participants could not perform certain assessments due to age, physical or health/medical conditions, and those parameters were omitted. Tests were performed with or without infusions. Adverse events were listed if they occurred. Anthropometric, anamnestic and disease-related information (eg, age, height, weight, diagnosis, number of exercise sessions and number of inpatient days before) were extracted from medical records.

Motor performance assessments, supervised by two exercise physiologists, were completed in the following order: static balance (1-min static stance/whole-body posture on a wooden bar, MOON test^24^), muscular endurance of the legs (5× sit-to-stand and MOON test^24^), hand grip strength (isometric maximum hand grip strength, Jamar hand-held dynamometer and MOON test^24^), leg extension strength (isometric maximum leg extension strength, self-conducted chair with digital force gauge, Sauter, Germany) and cycle ergometer test (endurance/exercise capacity on stationary bicycle ergometer and steep ramp test (SRT)^25 26^). Adequate recovery and hydration were provided. In the online supplemental file, assessments are presented in more detail.

10.1136/bmjsem-2024-001907.supp2 Supplementary data

For psychological parameters, HRQoL and fatigue were assessed. HRQoL was assessed using the short form 15 of the Paediatric Quality of Life Inventory (PedsQL SF15), measuring the core dimensions of physical, emotional, social and school functioning. Fatigue was assessed using the multidimensional PedsQL fatigue scale, which compromised general, sleep/rest and cognitive fatigue. The reliability and validity of the PedsQL SF15 and the paediatric multidimensional fatigue scale have been shown in paediatric cancer populations.^27^ Parents and participants completed this measure as a self-report and a parent report.

### Equity, diversity and inclusion

This study intentionally included participants based on gender, race/ethnicity/culture, socioeconomic level and representation from marginalised groups, with a commitment to high diversity. No individuals were excluded based on these factors. The investigator and author team is gender-balanced, comprising junior and senior researchers from multiple disciplines. Inclusive data collection methods were designed to accommodate diverse language backgrounds and age groups within the paediatric range, with no exclusions based on accessibility needs, regional differences, education or socioeconomic levels.

### Data analysis

Data analysis was done using SPSS (IBM SPSS Statistics V.27, Armonk, New York, USA), and correlation analysis and plotting were done using R (R Core Team 2023). The values of the motor performance test were adjusted to age-specific and gender-specific reference normative data by analysing the percentage difference between test results and normative data: $\frac{Differencebetweenthereferencevalueandmeasuredfitnessdata}{Referencevalue}\times 100.$ These values were calculated first individually before calculating the mean. For reference values, we collected data for each child (age-matched and gender-matched) in the 50th percentile for each test item in the literature. The results for static balance were compared with data by Bös *et al*,^27^ hand grip strength to data by Beenakker *et al*,^28^ leg extension strength to data by Nyström *et al*,^29^ steep ramp test to data by Bongers *et al*,^30^ sit-to-stand to unpublished age-specific and gender-specific student reference values (n=289),^26^ QoL (PEDS QL) to data by Engelen *et al* 31 and cancer-related fatigue (PEDS QL) to data by Varni *et al*.^24^ This is in line with other publications.^28–30^ A comparison of patients to differences from reference values for motor performance was performed using the Wilcoxon test as the non-parametric alternative due to the small groups. Psychological parameters were analysed with descriptive statistics. One SD from the mean score of the study population was set as a cut-off score to define an at-risk status in HRQoL and fatigue.^31^ Correlations were analysed using Spearman’s correlation coefficient r to examine associations between parameters. The fitness score comprises the mean values of all physical performance items (difference to reference). The endurance score is also calculated from the mean of the muscular endurance legs and the cycle ergometer test. The strength score consists of hand and leg strength and the motor performance score is static balance. To find out about predictors to aim for health-related risk factors and determine training recommendations, a generalised linear model was performed, where we looked at physical performance parameters and clinical data such as the number of supervised exercise sessions, the number of inpatient days, the days between diagnoses and baseline testing, age, body mass index (BMI) categories and gender. The level of statistical significance was set at p≤0.05.

## Results

### Participants

**Figure 1:**
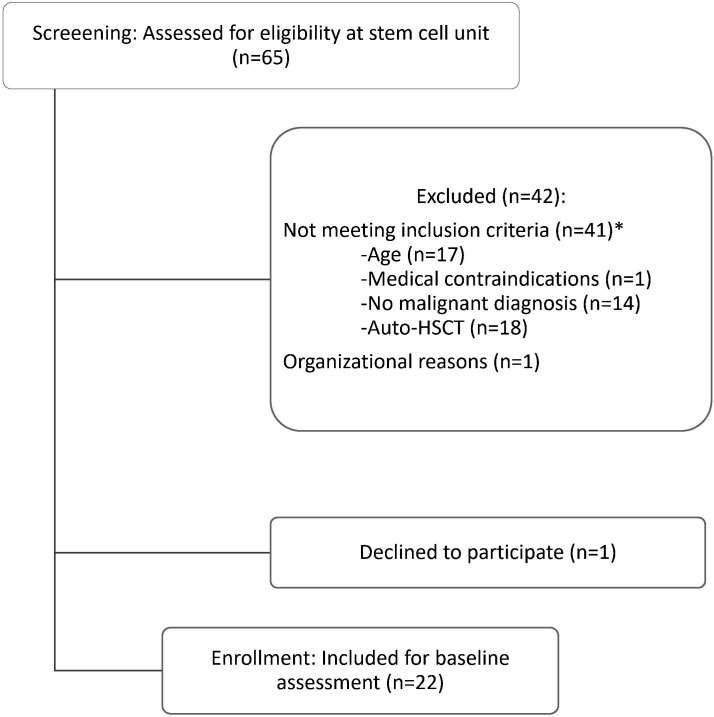
Recruitment diagram (Consolidated Standards of Reporting Trials). *In the following criteria, double designations are possible. HSCT, haematopoietic stem cell transplantation.

Between January 2020 and March 2023, 65 patients scheduled for stem cell transplantation were screened, with 23 meeting the inclusion criteria. Of these, 22 patients (96% recruitment rate) agreed to participate (Consolidated Standards of Reporting Trials diagram, figure 1). One teenager initially declined but later joined the usual care exercise programme. Five females (23%) and 17 males (77%) attended with a mean age of 9.36±4.53 years (median=7.50, range 4–18) and a BMI of 18.71±25.51. Categorised by percentiles (age-specific and gender-specific), BMI revealed three patients as underweight, 14 patients as healthy weight, two patients as overweight and three patients as obese; 16 patients were diagnosed with leukaemia (73%), two patients (9%) with myelodysplastic syndrome and neuroblastoma, and one (5%) child each with non-Hodgkin's lymphoma (NHL) and blastic plasmacytoid dendritic cell neoplasm (5%). Among the patients, 10 (45%) had relapsed diagnoses, with one experiencing a second one. Patients had 76.52±48.78 (median=66.00, range 7–221) inpatient days since diagnosis for HSCT and participated in 15.29±14.09 (median=11, range 1–52) supervised in-hospital exercise sessions before. One patient underwent a second haploidentical (haplo-) HSCT due to graft failure. Detailed patient characteristics are shown in table 1.

**Table 1:** Demographic and clinical characteristics of study patients before haematopoietic stem cell transplantation at baseline assessment

Characteristics	N (%)	Mean±SD	Median	Range
Age at inclusion (years)	22 (100)	9.4±4.5	7.50	4–18
Age and gender (years)				
Female	5 (23)	6.4±2.3	7	4–9
Male	17 (77)	10.2±4.7	10	5–18
Body mass index (kg/m²)	22 (100)	18.7±25.5	26.7	13–32
Cancer diagnosis				
Type of cancer				
ALL	8 (36)			
AML	8 (36)			
MDS	2 (9)			
Neuroblastoma	2 (9)			
BPDCN	1 (5)			
NHL	1 (5)			
Amount of relapsed cancer diagnosis within the patients	10 (45)	1.1±0.2	1	1–2
Days between diagnosis and assessment	22 (100)	174.3±79.7	157.5	67–432
Inpatient days until assessment	21 (95)	76.5±48.8	66.0	7–221
Supervised exercise sessions before assessment	21 (95)	15.3±14.1	11	1–52
Treatment protocols before assessment *				
AML-BFM	7 (32)			
ALL-Rez BFM	2 (9)			
AmoRe	2 (9)			
Experimental/individual	2 (9)			
INFORM	2 (9)			
EWOG-MDS	2 (9)			
ALL-BFM	1 (5)			
CoALL-Register	1 (5)			
EsPhALL	1 (5)			
IntREALL	1 (5)			
NHL-BFM	1 (5)			

*Treatment protocols in detail: ALL BFM (ALL BFM 2017), ALL-Rez BFM (2002), NHL-BFM (NHL-BFM Register 2012), AML-BFM (AML-BFM 2015, AML-BFM 2017, AML-BFM 2019, AML-BFM Register 2017), AMoRe2017, IntREALL 2010.

ALL, acute lymphoblastic leukaemia; AML, acute myeloid leukaemia; BPDCN, blastic plasmacytoid dendritic cell neoplasm; CoALL, Cooperative Study Group for Childhood Acute Lymphoblastic Leukemia; EsPhALL, European intergroup study of post-induction treatment of Ph-positive Acute Lymphoblastic Leukaemia; EWOG-MDS/SAA, European Working Group of Myelodysplastic Syndrome and Severe Aplastic Anemia in children and adolescents; INFORM, INdividualized Therapy FOr Relapsed Malignancies in Childhood; IntREALL, International Study for Treatment of Childhoof Relapsed ALL; MDS, myelodysplastic syndrome; NHL, non-Hodgkin lymphoma.

### Outcome analysis of baseline testing

Motor performance was measured in 22 children. The feasibility of motor performance tests concerning participation rates varied from 77% to 100%, with 21 (95%) participating in muscular endurance legs, 17 (77%) participating in static balance, 22 (100%) in hand grip strength, 19 (86%) in leg extension strength and 19 (86%) in the cycle ergometer test. Data collection of psychological parameters was feasible for 20 (91%) children and 18 (82%) parents in HRQoL and for 21 (95%) children and 18 (82%) parents in the fatigue questionnaire.

**Figure 2:**
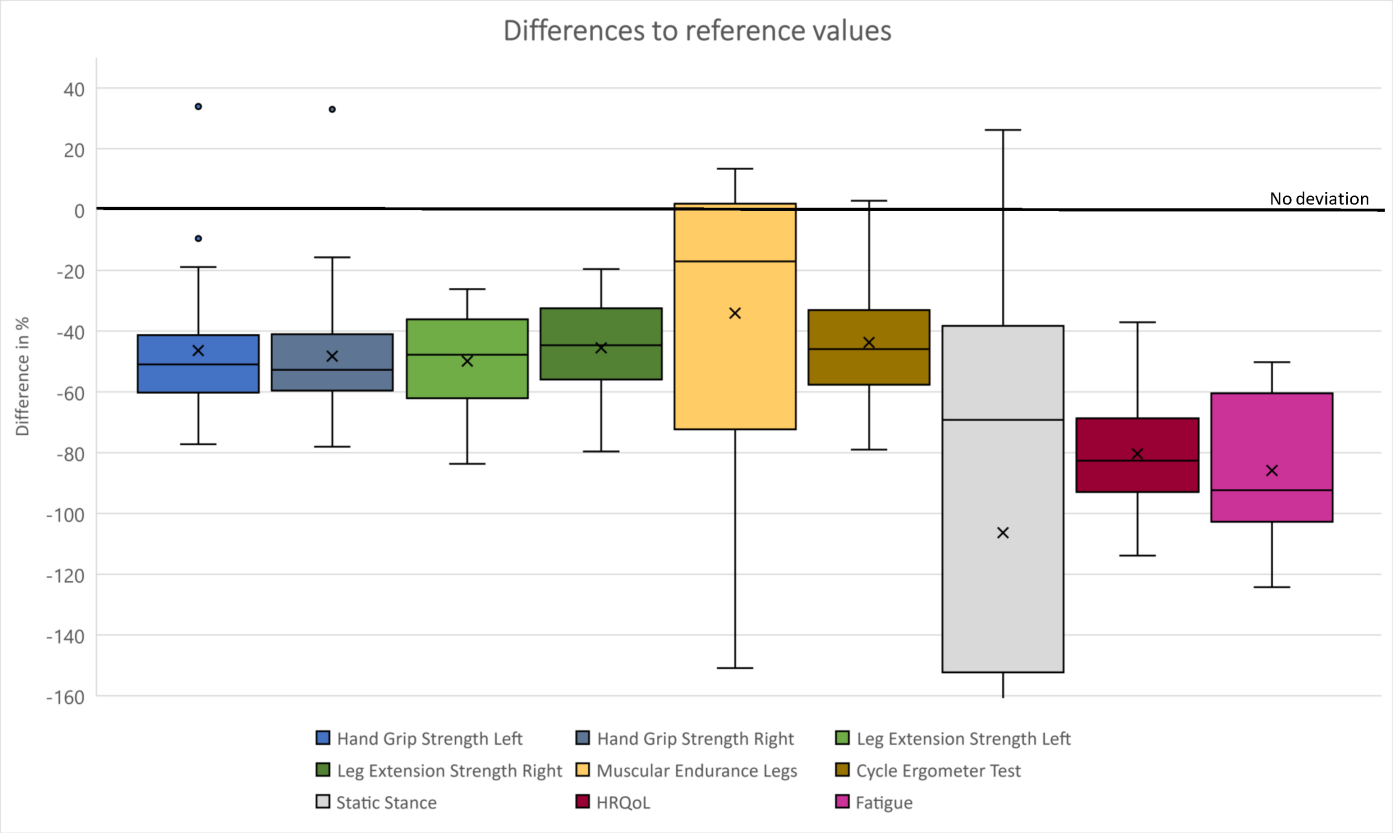
Differences in reference values.

The results for motor performance and psychological endpoints compared with healthy reference data are presented in table 2 and figure 2. The participants’ mean was significantly lower in all motor performance endpoints than the reference data of healthy age-specific and gender-specific values. For muscular endurance, participants scored −34% below reference values, which resulted in p=0.019. In static balance, they differed by −106% (p<0.001). For strength parameters, participants were significantly less in hand grip strength by −46% for the left and −48% for the right hand (left hand p<0.001 and right hand: p<0.001) and in leg extension strength by −50% for the left (p<0.001) and by −45% for the right leg (p=0.002). In the cycle ergometer test, they differed in peak W/kg body weight by −44% (p<0.001). In HRQoL, patients scored a significant difference of −80% (p<0.001) and differed in fatigue by −86% (p=0.016). Using one SD from the sample’s mean as a cut-off score, six children (27%) had a high-risk status in fatigue and four children (18%) in HRQoL. No exercise-related adverse events occurred during or after testing.

**Table 2:** Comparison of assessment results of the cancer patients to age-specific and gender-specific reference values from the literature

Motor ability	Test item		Baseline results			Mean of healthy age-specific and gender-specific reference values	P value	Mean difference to reference value (%)
		N (%)	Mean ± SD	Median	Range			
Muscular endurance legs	5× sit-to-stand (time s)	21 (95)	8.4±2.4	8.1	5.5–14.8	6.5	0.019*	−34±45
Static balance	Static stance (ground contacts)	17 (77)	16.6±8.3	18	4–30	8.9	<0.001***	−106±98
Hand grip strength left	Hand-held dynamometry (in kg)	22 (100)	11.9±10	7.8	3.2–39.1	19.3	<0.001***	−46±24
Hand grip strength right	Hand-held dynamometry (in kg)	22 (100)	13.6±11.7	8.6	3.2–46.7	22.1	<0.001***	−48±22
Leg extension strengthextension strength right Left	Force transducer (N)	19 (86)	117.5±67.6	110.7	24.2–283.5	239.7	<0.001***	−50±16
Leg extension strength right	Force transducer (N)	19 (86)	149.6±99.1	115.2	30.2–416.8	229.2	0.002**	−45±16
Cycle ergometer test	Steep ramp test (peak W/kg bodyweight)	19 (86)	2.9±0.9	3	1–4.8	5.2	<0.001***	−44±18
Quality of life†	PEDS QL, SF15	22 (100)	68.6±12.3	68.9	31.3–98.8	83.1	<0.001***	−80±19
Cancer-related fatigue†	PEDS QL, fatigue	22 (100)	72.1±15.2	74.3	44.4–100	84.2‡	0.016*	−86±21

Note: Sit-to-stand results were compared with unpublished age-specific and gender-specific student reference values (n=289).^42^ The results for static balance were compared with data by Bös *et al*.^43^ Hand grip strength was evaluated in relation to data provided by Beenakker *et al*.^44^ Leg extension strength results were compared with data from Nyström *et al*.^45^ The results of the steep ramp test were compared with previously published data from Bongers *et al*.^46^ Quality of life (PEDS QL) was compared with Engelen *et al* 47 and cancer-related fatigue (PEDS QL) to Varni *et al*.^27^

*The Wilcoxon test is significant at the 0.05 level (two-sided).

**The Wilcoxon test is significant at the 0.01 level (two-sided).

***The Wilcoxon test is significant at the 0.001 level (two-sided).

†Questionnaire scores were collected through patient self-reports (for those aged ≥8 years) and parental proxy reports (for those aged <8 years). In cases where no parental proxy report was available for participants under the age of 8, the patient's report was used.

‡Reference values were not age-specific and gender-specific due to a lack of data; they were adjusted to self-report or proxy reports.

PEDS QL, Paediatric Quality of Life.

### Correlation analyses

**Figure 3:**
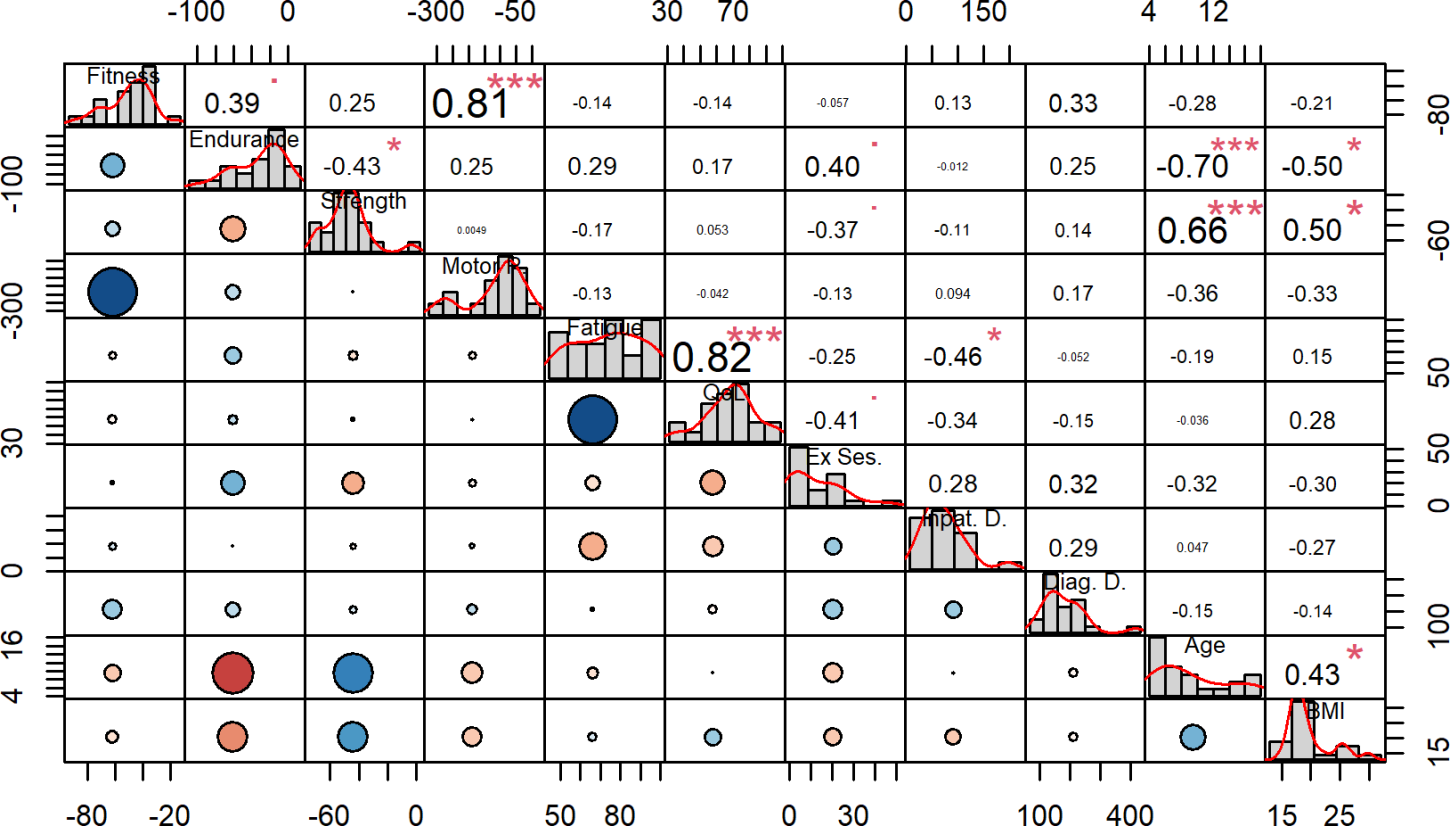
Pair panel correlation matrix. The figure displays the correlation coefficients between variables in matrix format, with a bar chart illustrating the distribution of parameters in the diagonal with their scales at the edges and correlation values presented in the upper off-diagonal cells (upper triangle). In the lower off-diagonal cells (panels on the lower triangle), the size and colour of circles indicate the strength (a bigger circle indicates a stronger correlation) and direction (blue positive and red negative) of the correlation. The labels for the columns and rows originate from the same parameter on the diagonal. The correlation matrix shows the following parameters: BMI, body mass index; Diag. D., days between diagnosis and assessment; Endurance, endurance/exercise capacity score; Ex Ses., number of supervised exercise sessions before assessment; Fatigue, fatigue score; Fitness, fitness score; Inpat. D., number of inpatient days until assessment; Motor P., motor performance score; HRQoL, health-related quality of life; Strength, strength score.

A pairs panel of the correlation analyses is shown in figure 3, in which summarised scores for fitness were used (all motor ability tests summarised), endurance/exercise capacity (cycle ergometer test and muscular endurance legs), strength (hand grip strength and leg extension strength) and motor performance (static stance). A correlation analysis was done for all variables. Correlations were found between fitness and motor performance scores (r=0.81, p<0.001). endurance/exercise capacity score correlates strongly negatively with age (r=−0.70, p<0.001) and BMI (r=−0.50, p=0.017). Strength score and age (r=0.66, p<0.001) and strength score and BMI (r=0.50, p=0.017) showed positive correlations. The analysis of the individual tests with the clinical data and exercise sessions (online supplemental material) reveals a negative correlation between supervised exercise sessions before testing and hand grip strength (r=−0.43, p=0.047), meaning less deviation of strength from reference values is related to more exercise sessions. Higher fatigue correlates moderately with more inpatient days (r=−0.47, p=0.034). A positive correlation between muscular endurance legs and the cycle ergometer test (r=0.7, p=0.002) and negative correlations between muscular endurance legs and hand grip strength (r=−0.51, p=0.019) and age (r=−0.73, p<0.001) were shown. Age and BMI have a positive correlation (r=0.43, p=0.045), and both show a correlation with hand grip strength (age: r=0.68, p<0.001; BMI: r=0.49, p=0.019), too. Between HRQoL and fatigue, there is a strong positive correlation (r=0.82, p<0.001). Both positively correlate with the cycle ergometer test (HRQoL: r=0.5, p=0.029; fatigue: r=0.53, p=0.019). The whole correlation matrix is available in the online supplemental material.

A generalised linear model was performed to determine predictors for health-related risk factors and to determine training recommendations. Only age (t=−2.183, p<0.05) seems to be a predictor in our model in terms of muscular endurance legs (t=−3.495, p<0.01) and in hand grip strength (t=2.757, p<0.05). The number of supervised exercise sessions seems to predict high fatigue (t=−2.407, p<0.05) and lower HRQoL (t=−3.763, p<0.01), which can be predicted by the number of inpatient days (−2.251, p<0.05), too (see online supplemental tables).

## Discussion

In this study, paediatric patients with cancer before allo-HSCT exhibited significantly reduced fitness compared with healthy individuals, aligning with previous research on paediatric patients with cancer without HSCT.^32–34^ Even before the initiation of chemotherapy, newly diagnosed paediatric patients with various cancer diagnoses demonstrated reduced physical capacity.^33^ However, most patients in this situation have traversed an extensive oncological therapy journey and are now embarking on a more intensive treatment, possibly further reducing their fitness levels.

Deisenroth *et al* 32 reported significant decreases in strength values across various muscle groups occurred approximately 36 days after primary treatment in children aged 5–18 years with different cancer entities. Their mean difference in leg extension was approximately −59%, even less than for participants in this study (right leg −36% and left leg −50%). Various factors could contribute to reduced strength, such as steroids, vincristine, reduced physical activity and neuropathic effects.^35^ Shoemaker *et al* 35 noted muscular weakness in paediatric patients with acute lymphoblastic leukaemia (ALL) and NHL, particularly within the initial 2 months of treatment, suggesting this weakness is often reversible. This may explain why participants in this study (mostly not currently receiving steroids and vincristine) demonstrated less loss. Ness *et al* 34 found significantly lower knee extension strength and hand grip strength in paediatric patients with ALL immediately after diagnosis, along with low bone mineral density, increased body mass, proximal muscle weakness, poor overall motor performance and low endurance. They also identified an impact of impaired knee extension strength on HRQoL.^34^ Notably, patients in this study reported reduced HRQoL and fatigue, common side effects in children and adolescents (6–19 years and 7–18 years) with mixed cancer entities.^36 37^ Still, we did not see an impact of knee extension strength on HRQoL in our patients. Compared with other studies, the participants of this trial had fatigue scores comparable with those of other patients with cancer (2–18 years) and were a bit below the mean score in HRQoL.^38^

The positive effect of the number of supervised exercise sessions before HSCT on hand grip strength supports the need for and beneficial effect of supervised exercise sessions during hospitalisation. Importantly, no exercise-related adverse events were observed during or after testing in our study, indicating the safety of our assessments, consistent with the literature.^39^

To gain a better understanding of how to support these children and adolescents facing cancer, we conducted an analysis of correlations to identify potential indicators for exercise interventions. We observed that as children get older and have a higher BMI, their exercise capacity tends to decrease, especially as shown by a negative correlation in muscular endurance in the legs. Age was also shown as a predictor of muscular endurance in the legs. This suggests the need for increased endurance and exercise capacity training, particularly among the older age group. Interestingly, there is an opposing effect when it comes to hand grip strength. Here, older participants show better results, as shown in regression analyses. That age seems to be an important factor, which aligns with Braam *et al*’s^40^ study of 60 children (8–18 years) with different entities. They revealed that children during or shortly after anticancer therapy were most inactive when having a higher fat mass, being fatigued, older and during treatment.

Additionally, we found that the duration of a child’s hospitalisation is linked to the number of supervised exercise sessions in which they participated. This implies that an extended hospitalisation provides more opportunities for supervised exercise sessions. However, it clarifies the association with poorer HRQoL and an increased level of fatigue, too. This highlights the importance of providing additional support and interventions for these patients, given the potential negative impact of extended hospitalisation on their well-being.

### Clinical implications

Poor fitness in children before HSCT shows the needo improve their fitness status. Children with lower fitness levels benefit from exercise programmes during HSCT,^23^ indicating potential for improvements. Additionally, initial data for adult patients undergoing HSCT suggests that exercise may improve survival and that higher baseline fitness levels are associated with lower mortality rates.^16^ Better pre-HSCT fitness is linked to improved overall survival.^17^ Therefore, enhancing pre-HSCT fitness status is recommended, potentially contributing to a better outcome for paediatric patients undergoing HSCT.

Our findings further indicate significantly reduced physical performance levels before paediatric HSCT, which carries several clinical implications. First, they underscore the need for developing prehabilitative interventions to stabilise physical well-being and HRQoL before the demanding treatment of HSCT. Enhanced physical fitness in patients may lead to better resilience to treatment and reduced side effects. Second, adolescent patients may benefit from targeted aerobic training, for example, on a stationary bicycle, due to their very low exercise capacity before HSCT. Third, our study suggests that supervised exercise sessions (comprising various child-adapted exercises) before HSCT are associated with better hand grip strength, highlighting the importance and feasibility of improving fitness before allo-HSCT. The design and methods of the exercise sessions were planned and conducted according to guidelines from the Network ActiveOncoKids.^40^ Additionally, the number of inpatient days before HSCT should be minimised whenever possible, as hospitalisation seems to be associated with higher fatigue levels, which can be minimised through exercises.^41^ Therefore, implementing prehabilitative strategies to enhance fitness may reduce side effect-related inpatient days and result in better HRQoL and less fatigue. A multidisciplinary team is recommended to optimally support patients, along with additional counselling and coaching for the entire family.

### Limitations

One limitation is the small patient sample, potentially hindering broader conclusions or generalisations. Missing data due to medical or psychological reasons is another limitation. The wide age range, while providing diversity, may confound variables affecting result consistency. Additionally, the gender imbalance restricts gender-specific assessments. Nevertheless, the study serves as a valuable validation of existing knowledge and offers practical insights for patient care. It highlights specific age groups, particularly older children, who may benefit from targeted training in certain areas. The study’s findings provide a foundation for developing training recommendations to enhance care for paediatric patients with cancer undergoing HSCT.

10.1136/bmjsem-2024-001907.supp1 Supplementary data

## Data Availability

Data are available upon reasonable request. Deidentified participant data are available upon reasonable request from the authors.
