# Supplementary material for: Severely reduced physical performance is already present at the time of admission for stem cell transplantation
Source: BMJ Open Sport Exerc Med. 2024 Jun 13;10(2):e001907. doi: 10.1136/bmjsem-2024-001907 (PMC11177700; doi:10.1136/bmjsem-2024-001907)
Supplement: Supplementary data [file bmjsem-2024-001907supp002.pdf]

Supplement Material

Motor performance assessments

Static Balance

The child tries to balance on his or her preferred leg on a T-bar for one minute with the other leg neither touching the floor, the bar nor the standing leg. The arms may be used for balancing. If the free leg touches the ground, time continues and the child should return to starting position as quickly as possible. If the child completely steps off the bar, or has longer contact with the ground, time is paused until back on starting position. Contacts to the floor are counted. If the free leg does not touch the ground for one minute, the task is solved perfectly. One run is performed with the preferred leg.

Muscular Endurance Legs

The child is sitting with a 90° knee and ankle angle on a stool with the arms crossed in the chest and asked to fully stand up and sit down again as quickly as possible for five times in a row. Three attempts are made to receive a mean value. Time is measured from the first movement of the patients until sitting down the fifth time.

Hand Grip Strength

The child sits in front of a table so that the elbow can be bent 90° and supported with a pad beneath on the table. The child holds the hand dynamometer in his/her hand and applies as much grip strength as possible. The width of the grip can be changed. Three tests are performed alternately for each hand, starting with the left hand.

Leg Extension Strength

The child sits on a high chair with a knee angle of 90°. The arms are crossed in front of the chest. There is no leaning. The left leg is fixed with a strap towards the back of the chair. In the strap is a force transducer. After a warm up of submaximal attempts, the patient is asked to slowly build up maximum isometric strength. Three attempts per leg are performed with a 30-second break between attempts.

Cycle Ergometer Test

Assessment of endurance/exercise capacity was performed by using the SRT on a stationary bicycle ergometer. After sitting for one minute on the ergometer to assess baseline data, the warm-up period starts, where the participant is asked to cycle at 60-80 rotations per minute (rpm) at 0W for three minutes. Then the test starts with an increase of 25W or 0.5W/kg bodyweight if under 30kg every 10s. If the participant is unable to keep above 50rpm, the test is stopped and is followed by 2-3 minutes cool-down of cycling at 0W. The heart rate at the end of each step, the maximum wattage achieved, the maximum heart rate, RPE scale and the total test time are recorded.

Correlation Analysis

		Muscular Endurance Legs	Cycle Ergometer Test	Hand Grip Strength	Leg Extension Strength	Static Balance	Fatigue	QoL	Amount of Supervised Exercise	Amount of Inpatient	Days Between Diagnoses	Age	BMI
Muscular Endurance Legs	Spearman's	—											
	p-value	—											
	N	—											
Cycle Ergometer Test	Spearman's	0.703 **	—										
	p-value	0.002	—										
	N	18	—										
Hand Grip Strength	Spearman's	-0.512 *	-0.161	—									
	p-value	0.019	0.508	—									
	N	21	19	—									
Leg Extension Strength	Spearman's	-0.018	0.304	0.458	—								
	p-value	0.945	0.235	0.050	—								
	N	19	17	19	—								
Static Balance	Spearman's	0.120	0.076	0.007	0.071	—							
	p-value	0.646	0.780	0.981	0.797	—							
	N	17	16	17	16	—							
Fatigue	Spearman's	0.262	0.533 *	-0.304	-0.021	-0.126	—						
	p-value	0.250	0.019	0.168	0.932	0.629	—						
	N	21	19	22	19	17	—						
QoL	Spearman's	0.070	0.502 *	-0.006	0.044	-0.042	0.823 ***	—					
	p-value	0.763	0.029	0.980	0.860	0.876	< .001	—					
	N	21	19	22	19	17	22	—					

Amount of Supervised Exercise Sessions	Spearman's	0.417	0.008	-0.427 *	-0.203	-0.130	-0.254	-0.413	—				
	p-value	0.060	0.974	0.047	0.405	0.620	0.254	0.056	—				
	N	21	19	22	19	17	22	22	—				
Amount of Inpatient Days	Spearman's	-0.270	-0.088	0.047	-0.022	0.094	-0.465 *	-0.342	0.282	—			
	p-value	0.250	0.729	0.838	0.934	0.730	0.034	0.130	0.216	—			
	N	20	18	21	18	16	21	21	21	—			
Days Between Diagnoses and Baseline Testing	Spearman's	0.176	0.089	-0.121	0.268	0.172	-0.052	-0.151	0.322	0.287	—		
	p-value	0.445	0.718	0.590	0.268	0.509	0.817	0.503	0.144	0.207	—		
	N	21	19	22	19	17	22	22	22	21	—		
Age	Spearman's	-0.729 ***	-0.390	0.677 ***	0.370	-0.357	-0.189	-0.036	-0.319	0.047	-0.151	—	
	p-value	< .001	0.098	< .001	0.119	0.159	0.401	0.874	0.147	0.838	0.502	—	
	N	21	19	22	19	17	22	22	22	21	22	—	
BMI	Spearman's	-0.362	-0.268	0.494 *	0.096	-0.333	0.154	0.278	-0.296	-0.267	-0.142	0.432 *	—
	p-value	0.107	0.265	0.021	0.694	0.191	0.493	0.211	0.180	0.242	0.527	0.045	—
	N	21	19	22	19	17	22	22	22	21	22	22	—

Note. * p < .05, ** p < .01, *** p < .001

Results of generalised linear model

Muscular Endurance Legs

Coefficients	Estimate	Std. Error	t value	P
(Intercept)	62.268423	26.114801	2.384	0.036 *
Amount of Supervised Exercise Sessions	0.401519	0.528330	0.760	0.463
Amount of Inpatient Days	-0.295034	0.139444	-2.116	0.058
Days Between Diagnoses and Baseline Testing	0.009091	0.075537	0.120	0.906
Age	-7.462189	2.134930	-3.495	0.005 **
BMI Categories: Obesity	-48.452878	19.540906	-2.480	0.031 *
BMI Categories: Overweight	0.528426	26.608942	0.020	0.98
BMI Categories: Underweight	-5.452307	17.424665	-0.313	0.760
Sex: male	-6.831654	18.978533	-0.360	0.726

*significant at the 0.05 level. **significant at the 0.01 level. ***significant at the 0.001. Null deviance: 39207.2 on 19 degrees of freedom. Residual deviance: 6006.3 on 11 degrees of freedom. Note: 2 observations deleted as missing, AIC: 190.85

Cycle Ergometer Test

Coefficients	Estimate	Std. Error	t value	P
(Intercept)	-0.63157	27.62056	-0.023	0.982
Amount of Supervised Exercise Sessions	-0.47192	0.61039	-0.773	0.459
Amount of Inpatient Days	-0.19857	0.14658	-1.355	0.209
Days Between Diagnoses and Baseline Testing	-0.02768	0.07275	-0.381	0.712
Age	-2.40126	1.96620	-1.221	0.253
BMI Categories: Obesity	-24.30000	18.80049	-1.293	0.228
BMI Categories: Overweight	-3.38605	24.86820	-0.136	0.895
BMI Categories: Underweight	-2.33189	16.53456	-0.141	0.891
Sex: male	15.68640	18.33638	0.855	0.414

*significant at the 0.05 level. **significant at the 0.01 level. ***significant at the 0.001. Null deviance: 6378.7 on 17 degrees of freedom. Residual deviance: 4127.9 on 9 degrees of freedom. Note: 4 observations deleted as missing, AIC: 168.91

Hand Grip Strength (Mean of left and right)

Coefficients	Estimate	Std. Error	t value	P
(Intercept)	-6.750e+01	1.233e+01	-5.473	0.000142 ***
Amount of Supervised Exercise Sessions	-3.419e-01	2.527e-01	-1.353	0.200999
Amount of Inpatient Days	6.095e-02	6.912e-02	0.882	0.395170
Days Between Diagnoses and Baseline Testing	9.558e-04	3.743e-02	0.026	0.980045
Age	2.753e+00	9.985e-01	2.757	0.017387 *
BMI Categories: Obesity	2.185e+00	9.606e+00	0.228	0.823859
BMI Categories: Overweight	-1.274e+00	1.312e+01	-0.097	0.924258
BMI Categories: Underweight	-8.090e+00	8.677e+00	-0.932	0.369511
Sex: male	-4.768e+00	8.916e+00	-0.535	0.602607

*significant at the 0.05 level. **significant at the 0.01 level. ***significant at the 0.001. Null deviance: 5014.5 on 20 degrees of freedom. Residual deviance: 1630.5 on 12 degrees of freedom. Note: 1 observations deleted as missing, AIC: 170.99

Leg Extension Strength (Mean of left and right)

Coefficients	Estimate	Std. Error	t value	P
(Intercept)	-42.68580	24.73515	-1.726	0.115
Amount of Supervised Exercise Sessions	-0.65868	0.61748	-1.067	0.311
Amount of Inpatient Days	-0.06927	0.13214	-0.524	0.612
Days Between Diagnoses and Baseline Testing	0.06798	0.07165	0.949	0.365
Age	-1.55570	1.92554	-0.808	0.438

BMI Categories: Obesity	-4.44655	18.18664	-0.244	0.812
BMI Categories: Overweight	32.19068	25.80731	1.247	0.241
BMI Categories: Underweight	7.69187	18.95254	0.406	0.693
Sex: male	15.69380	18.17761	0.863	0.408

*significant at the 0.05 level. **significant at the 0.01 level. ***significant at the 0.001. Null deviance: 6445.3 on 18 degrees of freedom. Residual deviance: 4762.8 on 10 degrees of freedom. Note: 3 observations deleted as missing, AIC: 178.88

Static Balance

**Coefficients**	**Estimate**	**Std. Error**	**t value**	**P**
(Intercept)	29.40968	174.97738	0.168	0.871
Amount of Supervised Exercise Sessions	-3.16509	4.03071	-0.785	0.458
Amount of Inpatient Days	0.45378	0.74333	0.61	0.561
Days Between Diagnoses and Baseline Testing	-0.07185	0.41665	-0.172	0.868
Age	-5.33625	12.55833	-0.425	0.684
BMI Categories: Obesity	-67.84532	124.99722	-0.543	0.604
BMI Categories: Overweight	-25.67549	153.79005	-0.167	0.872
BMI Categories: Underweight	-73.74079	91.82146	-0.803	0.448
Sex: male	-54.16394	105.24281	-0.515	0.623

*significant at the 0.05 level. **significant at the 0.01 level. ***significant at the 0.001. Null deviance: 156609 on 15 degrees of freedom. Residual deviance: 99267 on 7 degrees of freedom. Note: 6 observations deleted as missing, AIC: 205.13

Fatigue

**Coefficients**	**Estimate**	**Std. Error**	**t value**	**P**
(Intercept)	119.70617	17.17996	6.968	1.5e-05 ***
Amount of Supervised Exercise Sessions	-0.84716	0.352	-2.407	0.0331 *
Amount of Inpatient Days	-0.17509	0.09628	-1.819	0.0940
Days Between Diagnoses and Baseline Testing	-0.0264	0.05213	-0.506	0.6218
Age	-2.56216	1.39093	-1.842	0.0903
BMI Categories: Obesity	-17.66665	13.38088	-1.32	0.2114
BMI Categories: Overweight	23.03136	18.27565	1.26	0.2315
BMI Categories: Underweight	-8.52318	12.08685	-0.705	0.4942
Sex: male	10.9141	12.41982	0.879	0.3968

*significant at the 0.05 level. **significant at the 0.01 level. ***significant at the 0.001. Null deviance: 6825.9 on 20 degrees of freedom. Residual deviance: 3163.9 on 12 degrees of freedom. Note: 1 observations deleted as missing, AIC: 184.91

Quality of Life

**Coefficients**	**Estimate**	**Std. Error**	**t value**	**P**
(Intercept)	109.63643	13.27968	8.256	2.72e-06 ***
Amount of Supervised Exercise Sessions	-1.02393	0.27209	-3.763	0.0027 **
Amount of Inpatient Days	-0.16751	0.07442	-2.251	0.0439 *
Days Between Diagnoses and Baseline Testing	-0.03157	0.0403	-0.783	0.4486
Age	-2.1541	1.07516	-2.004	0.0682
BMI Categories: Obesity	-11.93322	10.34309	-1.154	0.2711
BMI Categories: Overweight	22.32601	14.12662	1.58	0.14
BMI Categories: Underweight	-8.75445	9.34283	-0.937	0.3672
Sex: male	17.54415	9.60022	1.827	0.0926

*significant at the 0.05 level. **significant at the 0.01 level. ***significant at the 0.001. Null deviance: 5768.5 on 20 degrees of freedom. Residual deviance: 1890.4 on 12 degrees of freedom. Note: 1 observations deleted as missing, AIC: 174.1
